# Quantifying DNA point mutations in commercially available AAV reporter vectors and plasmids

**DOI:** 10.1016/j.omtm.2025.101501

**Published:** 2025-06-02

**Authors:** Rebecca Rose, David J. Nolan, Jonathan A. DaRoza, Sanford L. Boye, Susanna L. Lamers

**Affiliations:** 1 BioInfoExperts, Alachua, FL 32615, USA; 2 University of Florida Powell Gene Therapy Center, Gainesville, FL 32605, USA

**Keywords:** AAV, single-genome amplification, sanger sequencing, genetic heterogeneity, quality control

## Abstract

To compare the accuracy of three sequencing methods for quantifying point mutations in AAV products, we sequenced the green fluorescent protein (GFP) transgene from two AAV9 reporter vectors (three lots each) and their respective plasmids, using three sequencing approaches: single-template amplification (STA) followed by Sanger sequencing (STA-Sanger) and two next-generation sequencing (NGS) platforms (Illumina and Oxford Nanopore Technologies [ONT]). Similar vector per-base mutation rates were found for both STA-Sanger (0.016%) and Illumina (0.013%), with slightly lower rates in plasmid sequences (STA-Sanger: 0.0019%; Illumina: 0.0074%), whereas ONT per-base mutation rates were much higher (vector: 2.2%; plasmid: 1.3%). The non-reference majority variant frequency (MVF) was more strongly correlated among vector lots for ONT (R^2^ = 0.91) compared with Illumina (R^2^ = 0.46), although correlation between plasmid and vectors was similar for both platforms (R^2^ = 0.23) and virtually non-existent between platforms for the same sample (R^2^ < 0.05). Overall, our results showed evidence for single-nucleotide mutations in AAV products, although there was a lack of consistency among sequencing platforms, which underscores the need for the AAV industry to develop sequencing methodologies with improved accuracy as a standard QC protocol.

## Graphical abstract

**Figure:**
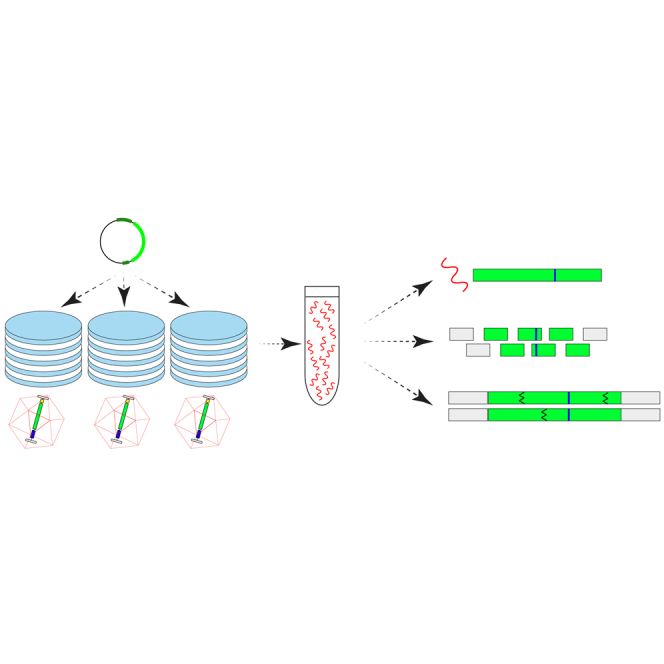

## Teaser

Rose and colleagues sequenced the transgene of commercially available AAV products using three different sequencing methods. Two methods showed a similar overall per-base mutation rate (consistent across suppliers and production lots) that was higher in the vectors than in the plasmids. However, there was little consistency among methods for identifying the specific location in the gene where the mutations occurred.

## Introduction

Adeno-associated virus (AAV) vectors are a popular gene delivery platform owing to specificity in tropism, reduced immunogenicity, limited integration potential, and long-term stable transgene expression.^1^^,^^2^^,^^3^ Seven AAV-mediated gene therapy products, primarily targeting rare diseases, have been approved by the Food and Drug Administration (FDA) to date. However, the field is experiencing explosive growth, and companies are increasingly developing AAV-based therapies for myriad genetic diseases. This expansion is exciting and potentially revolutionary; however, quality control of the manufactured products has not kept pace and remains an area for improvement.^4^^,^^5^

AAV products are subject to biological, physical, and chemical modifications during the production process,^6^ resulting in capsids that are completely empty, contain non-target DNA derived from producer cell and/or plasmid DNA, or contain the target AAV vector genome with major genomic rearrangements (e.g., truncations/chimeras) and/or single-nucleotide point mutations.^7^^,^^8^^,^^9^^,^^10^^,^^11^ Single-nucleotide mutations, especially in the transgene (the expected therapeutic), may ultimately result in a lack of transgene expression or sub-optimally performing gene product in the patient and contribute to safety risks.

While common AAV quality control (QC) assays address product defects and impurities, there is currently no standardized protocol for full genetic characterization of the manufactured AAV vector DNA.^12^ Advisory groups, including within the FDA, have noted that potentially toxic products could be pre-emptively identified through the development and adoption of better QC techniques, including next-generation sequencing (NGS), a relatively inexpensive and high-throughput method.^13^^,^^14^^,^^15^^,^^16^ NGS has been used in a handful of studies on AAV plasmids and vectors, and sequencing companies have introduced AAV-specific workflows.^11^^,^^17^^,^^18^^,^^19^^,^^20^^,^^21^^,^^22^^,^^23^^,^^24^^,^^25^^,^^26^ While these studies demonstrated the utility of NGS in identifying certain genetic features, e.g., truncations or chimeras, detecting single-point mutations is limited to a few studies using the Illumina^23^ and Oxford Nanopore Technologies (ONT)^20^ platforms. Furthermore, the AAV field has yet to adopt an industry-wide sequencing protocol, the use of which could largely eliminate inter-laboratory variability, increase confidence in this approach, and enable its use in federal regulatory standards.

To accomplish this, a “ground truth” for AAV vector sequences must first be established against which NGS-generated vector sequences can be compared. However, this is not necessarily straightforward. While the expected sequence from the parent plasmid is often used as the reference sequence, the actual plasmid used to seed a batch of AAV vectors may itself contain point mutations that originated during the manufacturing process (potentially requiring sequence verification for every plasmid lot). As point mutations may occur at many steps during the process of sequencing (e.g., DNA extraction, amplification, library prep, etc*.*), it is difficult to have 100% confidence of correctly disentangling production induced vs. experimentally induced mutations. One approach for attempting to derive the ground truth sequences is by using STA + Sanger sequencing (STA-Sanger). In this method, DNA is serially diluted and PCR-amplified to find the concentration at which only a single template is present in each reaction. Then, multiple PCRs, each containing a single template, are used to independently amplify the genetic region of interest, so that the end result is billions of copies of a single starting molecule. Each of the amplified products are sequenced using fluorescently labeled chain-terminating dideoxynucleotides and gel capillary electrophoresis (i.e., Sanger sequencing), which results in a chromatogram where each nucleotide in the sequence is represented by a different colored peak. Sites in the chromatogram that show two different colored peaks of approximately equal height (called “ambiguities”) indicate the presence of multiple nucleotides detected at that position, either because multiple templates were present in the original reaction or because of polymerase error during the first few rounds of PCR (mutations arising in subsequent rounds would be present at too low of a frequency to show up in the chromatogram). By identifying and removing ambiguities, polymerase error can largely be eliminated from the final dataset. This method has been used for decades by the HIV-1 field as the “gold-standard” sequencing approach for quantifying point mutations in a highly heterogeneous and high copy-number viral population.^27^^,^^28^ The disadvantage of this approach is that it is labor-intensive, expensive, and low-throughput. Furthermore, for a large population of virions, such as an AAV vector preparation, although it may be possible to estimate the mutational *depth* (i.e., the per-base mutation rate), it is unlikely to reflect the mutational *breadth* (i.e., the specific positions at which mutations occur.)

Here, our goal was to evaluate the performance of different sequencing methods for quantifying point mutations in the transgene of AAV plasmids and vectors. We used three sequencing protocols: STA-Sanger; Illumina, a high-throughput, short-read NGS platform in wide use across many applications; and ONT, a newer high-throughput, long-read NGS platform that is the most economical of the three tested here and can also be performed in-house. Although we initially planned to perform Pacific Biosciences single-molecule real-time (SMRT) sequencing as well, the relatively high DNA requirements for SMRT sequencing exceeded the total yield of our starting material. We sequenced the GFP gene in AAV vector products obtained from two different suppliers (three separate production lots of each product) and the corresponding producer plasmids. Our objective was to quantify and compare mutation rates among the products (vector vs. plasmid, vectors lots) and the platforms. Specifically, we sought to compare the per-base point mutation rate (1) between vectors and their associated plasmids, (2) among different lots of the same vector, and (3) among sequencing methods.

## Results

### No heat treatment and TE buffer as diluent reduced per-base point mutations/ambiguity rate in single-template amplicons of the GFP gene

**Figure 1:**
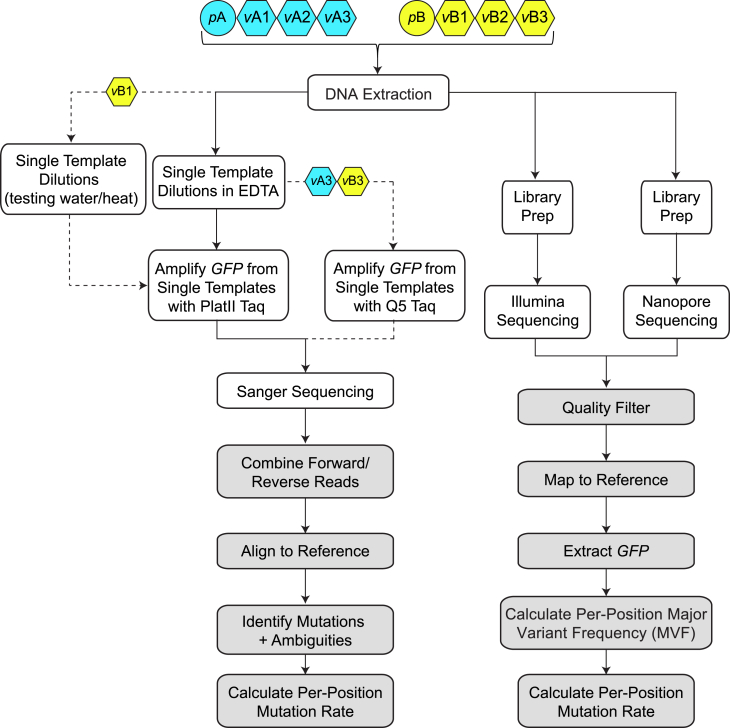
Flowchart of analysis Eight AAV samples were studied (top). All samples went through the main pipeline (denoted by solid arrows); a subset of samples had additional experiments performed (denoted by dotted arrows). White boxes denote experimental steps, and gray boxes denote analytical steps.

We purchased AAV vector and plasmid from two suppliers (Supplier A and Supplier B). The AAV vectors from both suppliers were packaged in AAV9, contained the green fluorescent protein (GFP) reporter gene, and were produced in adherent HEK293 cells. The products contained the chimeric CMV-chicken beta-actin promoter (CBA) and cytomegalovirus (CMV) promoters, respectively. DNA was extracted from three lots of vectors from each supplier (*v*A1*v*A3; *v*B1–*v*B3) as well as their respective plasmids (*p*A, *p*B) and used for library preparation and sequencing on the Illumina and ONT NGS platforms (Figure 1). In parallel, DNA from the same extraction was serially diluted to identify the concentration containing a single template, which were used to seed ∼75 independent PCRs for each sample to amplify the GFP gene. Each PCR product was then Sanger sequenced, and a consensus sequence of all the amplicons from each PCR was generated. Chromatograms were manually inspected to identify and remove ambiguities (Table 1).

**Table 1:** Point mutations and ambiguities in STA-Sanger sequences

Experiment^a^	Condition	Product	Total # seqs	Total bases	# Seqs with PM	# Seqs with AM	Total # PM	Total # AM	Per-base PM rate	Per-base AM rate
1	no heat/water (baseline)	*v*B1	41	29,520	12	8	29	17	0.098%	0.058%
	no heat/TE buffer^b^	*v*B1	78	56,160	7	10	17	16	0.030%	0.028%
	heat/water	*v*B1	28	20,160	17	4	41	8	0.203%	0.040%
2	PlatII	*v*A3	95	68,115	7	23	7	23	0.0092%	0.029%
	PlatII	*v*B3	102	73,440	5	17	6	18		
	Q5	*v*A3	83	59,511	1	0	1	0	0.0047%	0%
	Q5	*v*B3	95	68,400	4	0	5	0		
3	no heat/TE buffer/PlatII	*v*A1	84	60,228	10	9	12	9	0.016%	0.020%
		*v*A2	95	68,115	8	8	8	8		
		*v*B2	88	63,360	11	4	11	4		
		*p*A	79	56,643	0	0	0	0		
		*p*B	73	52,560	2	0	2	0		

a Experiment 1 = testing impact of heat/TE buffer; 2 = testing impact of Taq polymerase; 3 = generating sequences under established conditions.

b Conditions used for experiments 2 + 3. Underlined values indicate sequences in final dataset. AM = ambiguities; PM = point mutations.

We initially generated STA-Sanger sequences of the GFP gene (*n* = 147) from a single-vector product (*v*B1) to determine the effect on observed mutations of two independent variables related to the experimental setup: (1) the diluent (water vs. TE buffer) and (2) heat treatment on vector DNA. Our reasoning for testing the diluent was that both water and TE buffer are commonly used in DNA extractions, and potentially a source of inter-laboratory variability, although the higher pH and inclusion of EDTA in the TE buffer is expected to be more protective for the DNA. Our reasoning for using the heat treatment was to address the possibility that some proportion of the positive and negative sense single-stranded vector DNA molecules (derived from separate capsids) will anneal to each other, creating a mixed population of single-stranded and double-stranded molecules. If the annealed vector DNA molecules are not genetically identical, STA-Sanger will not accurately count vector DNA molecules with SNPs, as two different starting templates would be present during amplification. Heat treatment of the DNA prior to amplification could, in theory, de-nature the double-stranded products; however, heat treatment may also degrade the vector DNA, which could confound the accuracy of the results.

Compared to the baseline condition (water as a diluent and no heat treatment, *n* = 41 sequences), replacing the water with TE buffer in dilutions (and no heat treatment, *n* = 78 sequences) resulted in a reduction of the per-base mutation rate (0.098% vs. 0.030%) and per-base ambiguity rate (0.058% vs. 0.028%; Table 1). Compared to the baseline condition, the use of heat treatment (*n* = 28 sequences) resulted in an increased percentage of sequences with point mutations (0.098% vs. 0.20%) though a smaller decrease in the percentage with ambiguities (0.058% vs. 0.040%). Going forward, the TE buffer + no heat condition was used for all subsequent experiments.

### Q5 Taq polymerase eliminated all ambiguities, but not point mutations, in single-template amplicons of the GFP gene

To test our hypothesis that ambiguities (but not mutations) corresponded to the inherent error rate of the polymerase, we generated STA-Sanger sequences of one vector lot from each manufacturer (*v*A3, *v*B3) using a standard polymerase Invitrogen Platinum II (PlatII) and a more expensive high-fidelity polymerase New England Biolabs Q5 (Q5). Compared to sequences amplified with PlatII (*n* = 197), sequences amplified with Q5 (*n* = 178) showed a slight decrease in the per-base mutation rate (0.0092% vs. 0.0047%) but a complete elimination of ambiguities (0.029% vs. 0%), suggesting that the ambiguities (but not the mutations) were caused by polymerase error (Table 1).

### Per-base mutation rate was higher in single-template amplicons of the GFP gene in AAV vectors compared to plasmids

We generated STA-Sanger sequences for the remaining samples using PlatII, for a total dataset of 694 sequences (plasmids: *n* = 152; vectors: *n* = 542). Of these, 50 contained at least one point mutation (Table 1). Overall, more vector sequences contained a point mutation (*n* = 48, 8.9%) compared to plasmid sequences (*n* = 2, 1.3%), and the total number of point mutations was higher among all vector sequences (*n* = 61) compared to plasmid sequences (*n* = 2). The number of vector sequences in each lot with a point mutation ranged from 5 to 11, and both suppliers had a similar number of sequences with a mutation among the three lots combined (A: *n* = 23, B: *n* = 25). The average per-base error rate for GFP among vector lots ranged from 0.008%–0.030% (mean: 0.016%), whereas the average per-base error rate for GFP in the plasmids ranged from 0%–0.0038% (mean: 0.0019%; Table 2), Next, we examined the type of nucleotide mutations and their effect on the amino acid sequence. Because the GFP sequence differed by one amino acid between the companies, Supplier A and Supplier B sequences were analyzed separately. While we would have preferred to have identical gene sequences to compare, we were limited by GFP reporter assays with three available lots to compare, so we purchased the products with the most homology and which had three available lots for analysis. As expected, more changes were transitions than transversions (Supplier A: 89%; Supplier B: 72%; Table S1). Importantly, only 1/47 point mutations were a G > T transversion, which could have indicated DNA damage.^29^^,^^30^ For both suppliers, the majority of mutations resulted in a substituted amino acid (Supplier A: 70%; Supplier B: 64%). In addition, one sequence from both companies had a nucleotide mutation that resulted in a premature stop codon. Of the 63 point mutations, four were present at the same position in two separate sequences.

**Table 2:** Average per-base mutation rate in three sequencing platforms

Sample	STA-Sanger	STA-Sanger (mean)	Illumina	Illumina (mean)	ONT	ONT (mean)
*p*A	0.0000%	0.0019%	0.0073%	0.0074%	1.4%	1.3%
*p*B	0.0038%		0.0075%		1.2%	
*v*A1	0.020%	0.016%	0.011%	0.013%	2.2%	2.2%
*v*A2	0.012%		0.010%		2.1%	
*v*A3	0.010%		0.013%		1.9%	
*v*B1	0.030%		0.014%		2.3%	
*v*B2	0.017%		0.014%		2.5%	
*v*B3	0.008%		0.013%		2.5%	

### Average per-base mutation rate in illumina was >100× lower than ONT but comparable to STA-Sanger

NGS was performed on the same DNA extracted from the AAV vector products that were used in the STA-Sanger experiments. Overall, the Illumina runs produced an average of ∼6.3M reads, which was reduced to ∼2.2M after quality filtering (Table S2). For ONT, the runs produced an average of ∼122K reads, which was reduced to ∼70K after quality filtering. The number of filtered reads covering GFP ranged from 183,606 to 493,298 for Illumina and 1,604–113,191 for ONT, with an average quality score between 34 and 40 for Illumina and 28–37 for ONT. As expected for the long reads, the depth of read coverage was uniform across the GFP gene for ONT and more variable for Illumina (although always >20K for the latter; Table S2; Figure S1). The average per-base error rate for GFP was similar among all vector lots for the same platform (Illumina: 0.010%–0.014%; ONT: 1.9%–2.5%), although the difference *between* the two NGS platforms was several orders of magnitude (Table 2). Both plasmids showed a similar per-base error rate on the same platform, although again, the rates among platforms differed considerably (Illumina: 0.0073%–0.0075%; ONT: 1.2%–1.4%). Interestingly, the overall average rates for Illumina and STA-Sanger were similar for the vectors (STA-Sanger: 0.016%; Illumina: 0.013%) and plasmids (STA-Sanger: 0.019%; Illumina: 0.0074%).

### Median major variant frequencies were similar among vector lots and plasmid from the same supplier, but >50× higher in ONT vs. Illumina

**Figure 2:**
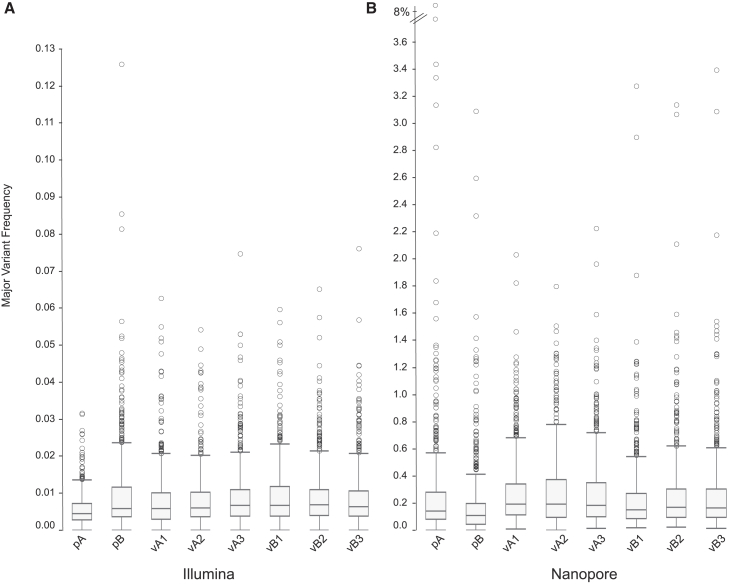
Boxplot of the majority variant frequencies for each position in GFP Majority variant frequencies (MVF) are shown for Illumina (A) and ONT (B). Boxes denote the IQR and median, and whiskers denote 1.5× the IQR range. Outliers are shown as open circles. Note the axis break in (B).

The major variant frequency (MVF, defined as the most prevalent non-reference nucleotide) was identified for all samples individually for each position in the GFP gene. For Illumina, the median MVF (over all GFP positions) was similar for the two plasmids (*p*A: 0.0044%; *p*B: 0.0058%) and for the vector lots (0.0058%–0.0068%; Figure 2A; Table S3). The IQR and ranges were also similar among all of the vectors. In contrast, for ONT, the median MVF was much higher for the plasmids (*p*A: 0.14%; *p*B: 0.11%) and the vectors (0.15%–0.19%; Figure 2B; Table S3), although again the IQR and ranges were similar among all six vector samples. (We note that the median/mean of the MVF is expected to be lower than the per-base mutation rate because the latter includes all variants at each site).

### Position-specific frequencies across GFP showed moderate correlation among lots from the same supplier but virtually no correlation for the same sample between the two platforms

Next, we looked at whether the MVF for each position was correlated among samples. Significant correlation was found among vector lots, and between vector and plasmid, in all cases (*p* < 0.0001). Among vector lots from the same supplier, the correlation coefficient was moderate for Illumina (Supplier A: R^2^ = 0.33–0.38; Supplier B: R^2^ = 0.56–0.60) and strong for ONT (Supplier A: R^2^ = 0.78–0.91; Supplier B: R^2^ = 0.98–0.99; Table 3; Figures S2 and S3). The correlation for MVF between plasmid and vectors was lower in all cases than among vector lots and was similar for both Illumina and ONT (Supplier A: R^2^ = 0.18–0.24; Supplier B: R^2^ = 0.33–0.38). On the other hand, there was no significant correlation in the MVF for the same sample between platforms (*p* > 0.05; Figure S4).

**Table 3:** R^2^ for plasmid vs. vector and vector vs. vector regression

Comparison^a^	Illumina R^2^	ONT R^2^
*v*A1 vs. *v*A2	0.33	0.79
*v*A1 vs. *v*A3	0.38	0.91
*v*A2 vs. *v*A3	0.34	0.78
*v*B1 vs. *v*B2	0.56	0.98
*v*B1 vs. *v*B3	0.57	0.98
*v*B2 vs. *v*B3	0.60	0.99
*p*A vs. *v*A1	0.18	0.22
*p*A vs. *v*A2	0.20	0.24
*p*A vs. *v*A3	0.18	0.23
*p*B vs. *v*B1	0.38	0.36
*p*B vs. *v*B2	0.37	0.34
*p*B vs. *v*B3	0.36	0.33

a All regressions were significant (*p* < 0.0001).

### Samples from the same company shared high-frequency positions more often in ONT data than in illumina data

We then examined, for each sample, the positions (*n* = 36) for which the MVF was among the highest 5% across all positions, referred to as MVF5 (Tables 4 and S4). In a perfect correspondence, all three vector lots and the associated plasmid from the same supplier would share the same 36 MVF5 positions. In the Illumina dataset, all three vector lots shared 15 MVF5 positions (Supplier A) and 19 MVF5 positions (Supplier B). Of these, the plasmid also shared 9 MVF5 positions (Supplier A) and 12 MVF5 positions (Supplier B). The number of shared positions among the vector lots was higher for ONT: all three vector lots shared 24 MVF5 positions (Supplier A) and 33 MVF5 positions (Supplier B). Of these, the plasmid also shared 18 MVF5 positions (Supplier A) and 14 MVF5 positions (Supplier B).

**Table 4:** MVF5 shared among vector lots and plasmids

Company	Samples	# Shared MVF5 positions (Illumina)	# Shared MVF5 positions (ONT)
A	3/3 vector lots^a^	15/36 (42%)	24/36 (67%)
	+ plasmid^b^	9/15 (60%)	18/24 (75%)
B	3/3 vector lots	19/36 (53%)	32/36 (89%)
	+ plasmid	12/19 (63%)	14/36 (44%)

a The denominator represents the highest possible number of shared positions among all three vector lots (i.e., all three vector lots have the same 36 MVF5 positions).

b The denominator represents the highest possible number of shared positions between the plasmid and the shared positions among the vector lots (numerator in the cell immediately above).

### Few positions were shared between platforms

We then looked at whether the same MVF5 positions were found in both Illumina and ONT for the same sample (Table S4). For all eight comparisons, only 1–4 MVF5 positions were shared cross-platform. All four Supplier A samples shared two MVF5 positions in both platforms (570 and 573), whereas the four Supplier B samples did not share any MVF5 positions in both platforms. However, all eight Supplier A and B samples shared four MVF5 positions (63, 532, 573, and 679) in the Illumina dataset and three MVF5 positions (319, 581, and 637) in the ONT dataset. Finally, we compared the location of point mutations in the STA-Sanger sequences to the MVF5 positions in the NGS data. Of the 63 point mutations in the STA-Sanger sequences, only 10 corresponded to MVF5 positions in at least one NGS dataset.

## Discussion

Manufactured AAV products are likely a mixture of (1) capsids with full-length vector genomes with complete fidelity to the parent plasmid sequence, (2) capsids that lack an AAV vector genome entirely, and (3) capsids that contain an AAV vector genome with imperfect fidelity to the parent plasmid sequence (including truncated genomes, chimeric genomes, and/or genomes with single-point mutation). Genomes with point mutations are the most difficult to characterize, since this requires a sequencing method that is both highly accurate (so that experimentally induced mutations are present at a much lower frequency than true mutations)^31^ as well as high-throughput, since a standard AAV vector preparation may contain trillions of individual vector genomes. This challenge is present for researchers in many fields characterizing highly heterogeneous populations,^32^^,^^33^ including quickly mutating viruses,^34^ microbiomes,^35^ and human somatic cancer mutations.^36^^,^^37^ Illumina short-read sequencing has high overall accuracy, but the structure of the AAV vector DNA is lost due to the fragmentary nature of the reads. Therefore, any measure of point mutations will be derived from all DNA molecules in the preparation, including from chimeric or truncated vector DNA genomes. Also, the mutation rate in incomplete DNA fragments (particularly near junctions) may differ from that in complete vector sequences. In contrast, long-read platforms such as those using nanopores can theoretically capture the entire length of the AAV vector genome in a single read; however, it is well known that these platforms have a high per-base error rate,^32^^,^^38^ which makes it extremely difficult to disentangle experimental error vs. true genetic heterogeneity.^31^ Long-read circular consensus sequencing (CCS) offered by Pacific Biosciences improves the accuracy of SMRT sequencing by deriving a consensus sequence from multiple passes of a single-template molecule^39^ and has been used to characterize the DNA cargo of AAV products^17^^,^^18^^,^^40^^,^^41^; however, none of these studies specifically measured the SNP rate of the vector DNA or compared the CCS SNP rate with other technologies, and SMRT sequencing requires an order of magnitude more starting product than either Illumina or ONT,^42^ which, along with the cost, can render this approach impractical. STA-Sanger is a labor-intensive, expensive, and low-throughput method; however, it is used by other fields as the “gold-standard” sequencing approach for quantifying point mutations in a highly heterogeneous and high copy-number viral population^27^^,^^28^ because when amplifying a single starting template, polymerase errors will be detected in the final consensus sequence as ambiguities and readily identified and removed. In this study, we compared the performance of three different sequencing platforms (STA-Sanger, Illumina, and ONT) for quantifying point mutation rates in the GFP gene in vectors/plasmids obtained from two different suppliers. While we intended to include SMRT as well, vector lots produced insufficient DNA to complete this. Future studies will ideally include all three NGS platforms.

First, we generated >500 STA-Sanger sequences to perform preliminary investigations into whether experimental conditions (water vs. TE buffer as diluent and heat treatment to denature annealed single-stranded template prior to amplification) and/or choice of Taq polymerase (PlatII vs. Q5) impacted the observed mutations and/or ambiguities. We found that heat treatment and water increased the mutation rate 7-fold compared to no heat/TE buffer, suggesting that these conditions degraded and/or damaged the single-stranded DNA prior to amplification, such that changes to the DNA sequence appeared as full mutations in the final consensus sequence. On the other hand, while Q5 resulted in a slightly lower mutation rate than PlatII, it completely eliminated all ambiguities, consistent with the hypothesis that the higher-fidelity Q5 introduced fewer errors during amplification. Furthermore, the per-base ambiguity rate in our STA-Sanger sequences was ∼2 × 10^−4^, similar to the per-base error rate of SGA^43^ and Taq polymerase^44^^,^^45^ reported previously, providing further evidence that the Platinum II Taq was working as expected. By removing sequences that contained ambiguities, we have greatly reduced the chance that point mutations detected in vector DNA using STA-Sanger were the result of polymerase error, rather than reflective of true genetic diversity in the viral sequences.

Interestingly, we found that the overall per-base mutation rates in the GFP gene were remarkably similar between the Illumina and STA-Sanger datasets for vectors (0.013% and 0.016%, respectively). Per-base mutation rates were lower in plasmids compared to vectors for both STA-Sanger and Illumina (0.0019% vs. 0.0074%, respectively). In contrast, the ONT per-base mutation rates were several orders of magnitude higher than STA-Sanger and Illumina. ONT is known to have a higher sequencing-induced error rate than Illumina, but the extent of the difference was still surprising. Future studies are ongoing to quantify the rate in other parts of the vector genome (e.g., the promoters) and in constructs with different reporter genes.

We note that this study was intended to compare the performance of the different sequencing platforms/approaches, and while the similarity in mutation rates between the two very different STA-Sanger and Illumina AAV vector datasets is encouraging, clearly, it would be advantageous to have a “ground truth” baseline against which to compare different approaches. In practice, however, this is not straightforward. Although STA-Sanger is a highly accurate method, the practical limitations of the method result in relatively few sequences compared to the trillions (or more) of virions in the AAV vector preparation, and stochastic effects will have a greater impact—as observed here by the higher variation among the vector lots in the STA-Sanger sequences compared to Illumina. It is possible that the impact could be mitigated by increasing the number of STA-Sanger sequences for a given sample, and we intend to investigate this further in the future. Additionally, although this method should be able to distinguish between amplification-induced errors and true mutations in the template, it is of course possible that post-transfection factors, including harvest, purification, storage, product handling, and/or DNA extraction, could damage the template prior to the amplification step. The use of the plasmid sequence as the “gold standard” is also potentially problematic, as we showed here that while the mutation rate was much lower in the plasmids compared to the vectors, the plasmid sequences recovered were not identical to the expected sequence. It is possible that these mutations arose at some point during the experimental process; on the other hand, these mutations could have been present in the plasmids prior to transfection. Understanding the plasmid heterogeneity will be important to disentangle plasmid polymorphisms from those found in vector DNA, which may have been introduced during the manufacturing process due to mistakes in the replication of vector DNA by biological machinery of the producer cells. In the future, it would be desirable to sequence each aliquot of plasmid that is used to seed a given vector lot, which unfortunately we were unable to do here. Additionally, future experiments could remove the ITR regions using restriction enzymes in both the vector and the plasmid DNA prior to the amplification step, which would eliminate potential secondary-structure interference with the amplification/sequencing process.

Surprisingly, given the difference in mutation rates between Illumina/STA-sanger vs. ONT, we found that the MVF for all GFP positions, as well as the highest frequency MVF positions (MVF5), were more strongly correlated among vector lots for the ONT datasets as compared to the Illumina datasets (i.e., intra-platform comparisons), although the correlation between vectors and plasmids was weak for both platforms. This might suggest that there is signal in the ONT data for true mutations that can be detected across vector lots, or this could also suggest that some chemical or physical property of the DNA is driving the shared patterns. We also found that all of the vectors and plasmids from both suppliers shared the same three MVF positions in the Illumina dataset and the same four MVF positions in ONT (although these positions were different between the platforms). There are well-known DNA characteristics that affect, to some degree, sequencing accuracy across all platforms, although the impacts may be more pronounced for some combinations (e.g., GC-context particularly affects short-read platforms; long stretches of homopolymers or dinucleotide repeats particularly affect nanopore sequencing) and the effects may manifest differently (e.g., insertions or deletions are more often found in sequences from long-read platforms as compared to short-read platforms).^46^^,^^47^^,^^48^^,^^49^ We did not observe any of these characteristics in the sequences at the location of the putative mutations; nonetheless, as it is unlikely that products from two different suppliers would have the same mutations, this further suggests an inherent bias in the sequencing platform (although these could have arisen by chance as well). Furthermore, there was virtually no correlation between MVF in the same sample when comparing Illumina vs. ONT, drawing into question how reliable these platforms are, particularly for detecting mutations at specific positions.

To improve the accuracy of the NGS methods, unique molecular identifiers (UMIs) could be used to tag individual vector DNA molecules prior to amplification/sequencing, which could enable the elimination of experimentally induced mutations through consensus sequence reconstruction.^35^^,^^50^ This technique has been used with success in other applications, such as human cancer somatic mutations. Another option could be the use of rolling circle amplification,^32^^,^^37^^,^^51^ similar to the CCS approach (although applicable to any platform), which could also help eliminate experimentally induced errors. The impact of high-fidelity polymerases in the library preparation steps could be explored, as well as the elimination of amplification steps altogether. Additionally, to investigate both the accuracy and the sensitivity of NGS methods, future experiments could include an AAV with a known mutation in the transgene that is spiked into the plasmid prep at a variety of dilutions (e.g., 10%, 1%, 0.1%, etc.). This would help to determine if the frequency of mutations in the NGS dataset is proportional to the actual frequency and detect the frequency threshold at which real mutations are indistinguishable from sequencing errors.

Overall, the similarity in mutation rate in AAV vector products purchased from two different suppliers, among different production lots, and by using two very different sequencing methods, suggests that there may be real underlying mutations. Our results also highlight that, while promising, current off-the-shelf library construction and NGS sequencing protocols do not appear to be sufficient for assessing genetic diversity in AAV with the high confidence required for use in regulatory settings.

## Materials and methods

### Vectors and plasmids

Three separate lots (produced on different dates) of GFP reporter assays were purchased from two different suppliers, as well as a representative aliquot of the producer plasmid. During manufacturing, producer plasmid DNA was co-transfected into adherent HEK293 cells with helper plasmids using supplier-specific and proprietary conditions. Cell lysate was purified using discontinuous iodixanol/cesium chloride density gradient centrifugation. Purified vector was concentrated and buffer exchanged. Final vector preparations were aliquoted and stored frozen at −80°C. Aliquots (∼4 × 10^12^ vector copies in 100 μL) were purchased and transported on dry ice to the sequencing facility.

### Single-template amplification

DNA was extracted from the vectors using the Invitrogen PureLink Viral RNA/DNA Mini Kit (#12280050) and quantified via Qubit. Nested PCR primers were designed using Geneious Prime for each of the constructs to amplify a region that included the entire GFP gene (Table S5). DNA dilutions were prepared to perform STAs, using 10 mM Tris and 0.1 mM EDTA pH 8.0 (Integrated DNA Technologies 1× TE Solution, #11-05-01-09). During early optimization experiments, water was also used as a diluent and heat treatment utilized to minimize the possibility of double-stranded vector DNA molecules (95°C for 10 min; hold at 4°C). Invitrogen Platinum II Hot-Start Green PCR Master Mix (#14001014) with GC enhancer was used to amplify the vector DNA in a 25 μL reaction volume with 0.2 μM final primer concentrations. PCR conditions for Supplier A products was 94°C initial denaturation for 1 min, then 35 cycles of 94°C for 15 s, 58°C primer annealing for 30 s, and 68°C extension for 1 min, followed by a final extension for 10 min at 68°C for both first and second round PCRs, whereas Supplier B products used a similar PCR cycling protocol but with a 60°C annealing temperature. After nested PCR amplification, gel electrophoresis was conducted with 1% agarose gels in 1× TAE buffer to visualize PCR products; PCRs with visible product on the gel after nested PCR are considered positive. DNA in a range of serial dilutions for each lot/sample was amplified to identify the dilution at which ≤30% of PCR wells were positive, indicative of 80% probability of a single starting template.^28^

### Single-template sequencing

Using the single-template dilution, positive PCR reactions were generated and sent to Azenta for Sanger sequencing. Chromatograms were assembled and consensus sequences extracted and trimmed to include only the 717/720bp GFP gene for C1/C2, respectively, using Geneious Prime v. 2024.1 software. After alignment to the reference sequence, all point mutations and ambiguities were confirmed through a manual review of the Sanger sequence chromatograms. To evaluate the fidelity of the initial polymerase (PlatII), we also used a high-fidelity polymerase (Q5) for an additional 178 sequences (New England Biolabs Q5 Hot Start High-Fidelity DNA Polymerase #M0494). Geneious Prime v. 2024.1 software was used to assess the type (transition/transversion), effect (amino acid change/no amino acid change/stop), and location of the point mutations. Per-base mutation rates were calculated as the total number of point mutations among all sequences divided by the total number of bases analyzed (i.e., the GFP gene length multiplied by the number of sequences).

### Next-generation sequencing

To determine whether NGS would show similar results to the STA-Sanger, we used the same extracted DNA for Illumina and ONT sequencing. Vector DNA was heat annealed to create double-stranded molecules of positive and negative sense vector DNA by heating the DNA to 95°C for 5 min, then ramping the temperature down by 1°C per minute to 25°C.^40^ Illumina sequencing libraries were prepared using the tagmentation-based Illumina DNA Prep kit and custom IDT 10bp unique dual indices (UDI) with a target insert size of 280 bp. No additional DNA fragmentation or size selection steps were performed. Illumina sequencing was performed on an Illumina NovaSeq X Plus sequencer producing 2 × 151 bp paired-end reads. Demultiplexing, quality control, and adapter trimming were performed with *bcl-convert1* (v4.2.4). ONT sample libraries were prepared using the PCR-free ONT Ligation Sequencing Kit (SQK-NBD114.24) with the NEBNext Companion Module (E7180L) to the manufacturer’s specifications. No additional DNA fragmentation or size selection was required. ONT sequencing was performed on a MinION Mk1B using R10.4.1 flow cells. Dorado (v0.9.1) was used in the super-accurate base calling mode. NGS sequencing was performed by SeqCenter, LLC.

### NGS analysis

To reach maximum data integrity, we filtered raw reads using a stringent threshold, filtering out reads with mean quality below 15 (ONT) or 38 (Illumina) and either <100 bp (Illumina) or >10,000 bp (ONT). Filtered reads were then mapped to the plasmid reference sequence obtained from the respective companies using *bwa-mem*^52^ and *minimap2*^53^ for Illumina and ONT, respectively. After mapping, Illumina reads had 10 bp removed from each side using *bamUtil*^54^ to account for lower confidence placement of these bases in the mapping alignment. Bam files were filtered to include just the GFP gene using *bamUtil* for Illumina data and *samtools*^55^ for ONT. Mapping statistics were analyzed using *samtools stats*^55^ where per-base rates were calculated as the number of mismatches/total bases mapped. Single-nucleotide variants were called using *bcftools*, and the non-reference majority variant (MV) was defined as the alternative allele found in the most reads at a given position, and the MV frequency (MVF) was calculated as the number of reads with the MV divided by the total number of reads (number of reads with the reference allele + number of reads with any other non-reference variant). As the GFP sequence was different between the two companies, positions in the GFP gene were shifted by three nucleotide positions for Supplier A so that both were 720 bp when comparing specific positions. High-frequency MVFs were defined as frequencies in the upper 95% percentile for each sample (vector lots from each supplier were averaged together first). Linear regressions were performed in R.

## Data availability

AAV sequences have been deposited to the Sequence Read Archive (SRA) under the accession numbers SRA: SRR32669214–SRR32669229 (BioProject: PRJNA1235260). STA-Sanger sequences were deposited in GenBank under accession numbers GenBank: PV365348–PV366288.

## Acknowledgments

S.L.L. was funded by Food and Drug Administration Small Business Innovative Research Award #1R43FD008039-01.

## Author contributions

R.R., writing (lead), formal analysis (equal), conceptualization (equal), investigation (equal), validation (equal), and methodology (equal). D.J.N., conceptualization (equal), investigation (equal), methodology (equal), validation (equal), formal analysis (equal), and writing (supporting). J.D., investigation (supporting) and validation (supporting). S.L.B., methodology (supporting) and writing (supporting). S.L.L., funding acquisition (lead), supervision (lead), conceptualization (equal), investigation (equal), and writing (supporting).

## Declaration of interests

R.R., D.J.N., J.D., and S.L.L. are employed by BioInfoExperts, LLC.

## Supplemental information

Document S2. Tables S1 and S4

Document S3. Article plus supplemental information
