# Supplementary material for: Quantifying DNA point mutations in commercially available AAV reporter vectors and plasmids
Source: Mol Ther Methods Clin Dev. 2025 Jun 2;33(3):101501. doi: 10.1016/j.omtm.2025.101501 (PMC12206035; doi:10.1016/j.omtm.2025.101501)
Supplement: Document S1. Figures S1–S4 and Tables S2, S3, and S5 [file mmc1.pdf]

OMTM, Volume *33*

Supplemental information

Quantifying DNA point mutations in commercially available AAV reporter vectors and plasmids

Rebecca Rose, David J. Nolan, Jonathan A. DaRoza, Sanford L. Boye, and Susanna L. Lamers

Table S1. Point mutations in the STA-Sanger sequences. See Excel file.

Table S2. Results from NGS analysis.

Platform	Sample	Raw Reads	Filtered Reads	Primary Reads Mapping	# Reads in GFP	Average Quality
Illumina	pA	10,337,694	2,519,448	2,446,547	348,447	34.3
Illumina	vA1	5,086,401	2,797,160	2,768,631	183,606	40
Illumina	vA2	3,888,452	1,839,336	1,818,534	269,582	40
Illumina	vA3	6,196,033	2,444,624	2,418,017	324,023	40
Illumina	pB	9,677,778	2,672,302	2,626,723	349,739	34.4
Illumina	vB1	4,506,534	1,520,434	1,518,078	413,391	40
Illumina	vB2	5,786,363	1,831,072	1,827,717	493,298	40
Illumina	vB3	5,472,176	1,794,876	1,792,219	475,291	40
ONT	pA	133,279	40,640	23,506	5,588	37.2
ONT	vA1	181,594	68,940	68,377	7,961	28.1
ONT	vA2	14,180	5,642	5,363	1,604	28.1
ONT	vA3	40,354	17,508	17,007	5,640	28.1
ONT	pB	47,046	27,454	16,867	3,793	37.7
ONT	vB1	148,581	108,651	105,951	75,186	29.1
ONT	vB2	228,370	160,072	158,324	113,191	29.0
ONT	vB3	185,587	129,319	127,080	89,357	29.0

Table S3. Percent MVF median/IQR among samples.

Platform	Sample	Median	IQR	Range
Illumina	pA	0.0044	(0.0027 - 0.0070)	(0 - 0.032)
	pB	0.0058	(0.0036 - 0.012)	(0 - 0.13)
	vA1	0.0058	(0.0029 - 0.010)	(0 - 0.063)
	vA2	0.0060	(0.0035 - 0.010)	(0 - 0.054)
	vA3	0.0066	(0.0038 - 0.011)	(0 - 0.075)
	vB1	0.0067	(0.0037 - 0.012)	(0 - 0.060)
	vB2	0.0068	(0.0040 - 0.011)	(0 - 0.065)
	vB3	0.0064	(0.0037 - 0.011)	(0 - 0.076)
ONT	pA	0.14	(0.078 - 0.28)	(0 - 8.2)
	pB	0.11	(0.043 - 0.19)	(0 - 3.1)
	vA1	0.19	(0.11 - 0.34)	(0.01 – 2.0)
	vA2	0.19	(0.095 - 0.37)	(0 - 1.8)
	vA3	0.18	(0.097 - 0.35)	(0.014 - 2.2)
	vB1	0.15	(0.083 - 0.27)	(0.018 - 3.3)
	vB2	0.17	(0.092 - 0.30)	(0.021 - 3.1)
	vB3	0.16	(0.093 - 0.30)	(0.016 - 3.4)

Table S4. MFV5 positions shared by groups of samples. See Excel file.

Table S5. Primers Used for STA-Sanger sequencing.

Sample	Step	Direction	Primer Sequence 5'-3'
Company A	PCR Round 1	Forward	CCTCTGCTAACCATGTTCAT
Company A	PCR Round 2	Forward	GCAACGTGCTGGTTATTGT
Company A	PCR Round 1	Reverse	GCAATAGCATCACAAATTTCAC
Company A	PCR Round 2	Reverse	GTGGTTTGTCCAAACTCATC
Company B	PCR Round 1	Forward	GGCAACTAGAAGGCACAGT
Company B	PCR Round 2	Forward	GGCAAACAACAGATGGCTG
Company B	PCR Round 1	Reverse	CGACTCACTATAGGGAGAC
Company B	PCR Round 2	Reverse	CCCACTGCTTACTGGCTTA

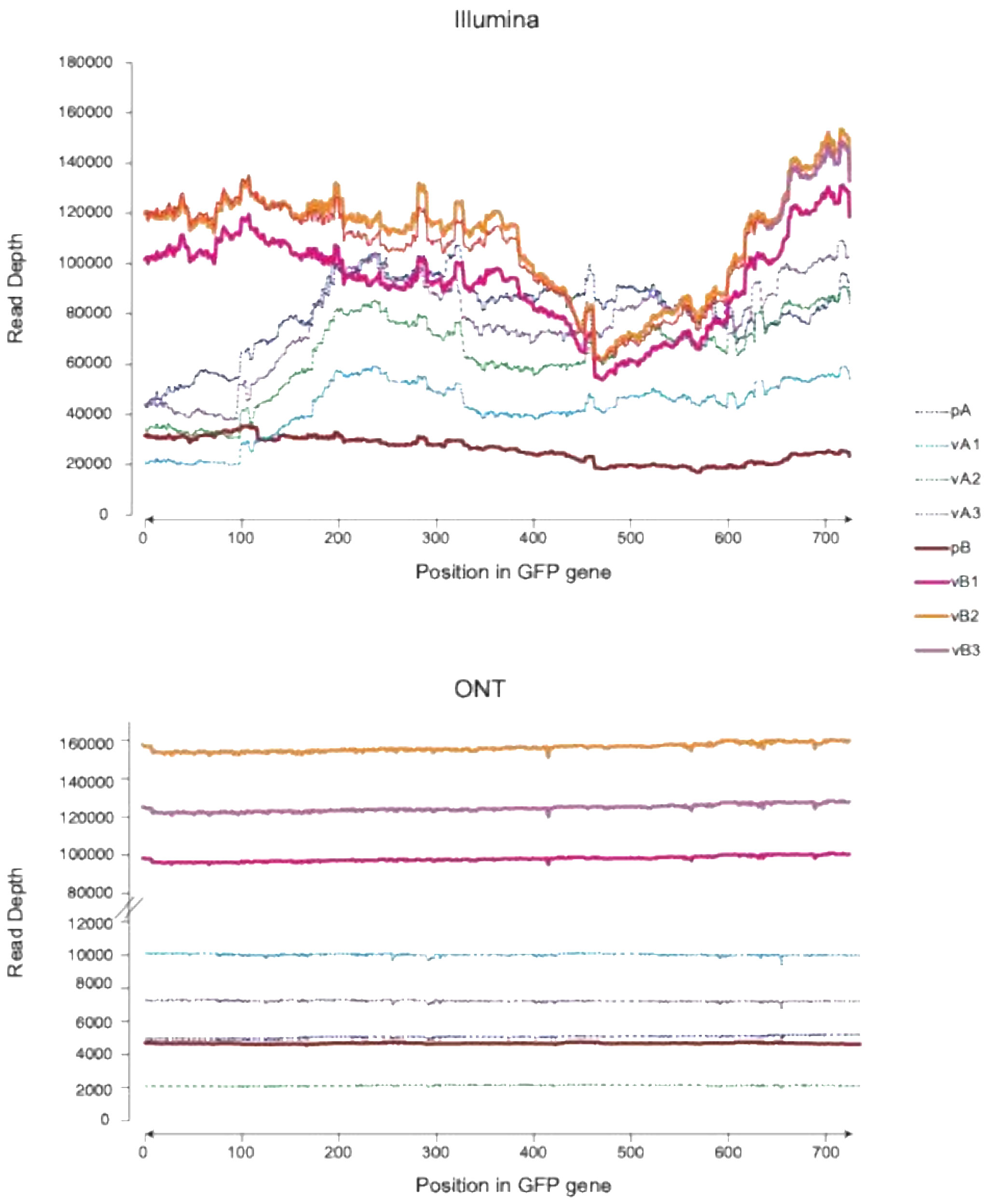

Figure S1. Depth of coverage across GFP for Illumina (top) and ONT (bottom). Samples are denoted by line color and supplier according to the legend (Supplier 1: dotted thin lines; Supplier 2: solid thick lines).

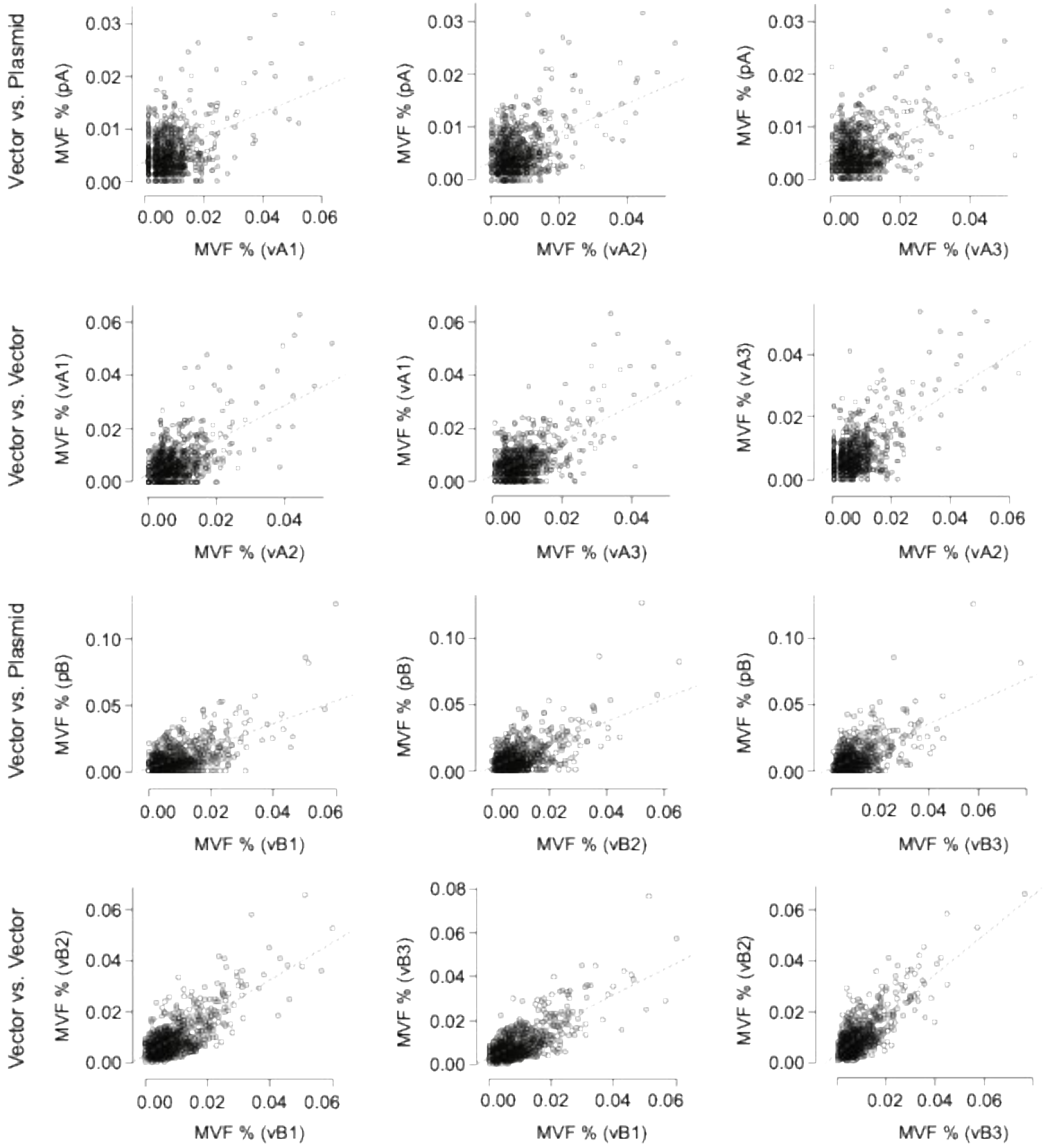

Figure S2. Scatterplot of MVF (%) from Illumina between vector and plasmid or between vector lots for Supplier A (top) and Supplier B (bottom). Dots indicate the MVF frequency (%) for each position in the GFP gene. A linear regression is shown as a dotted line. R^2 values are given in the text; all correlations were significant (p<0.0001).

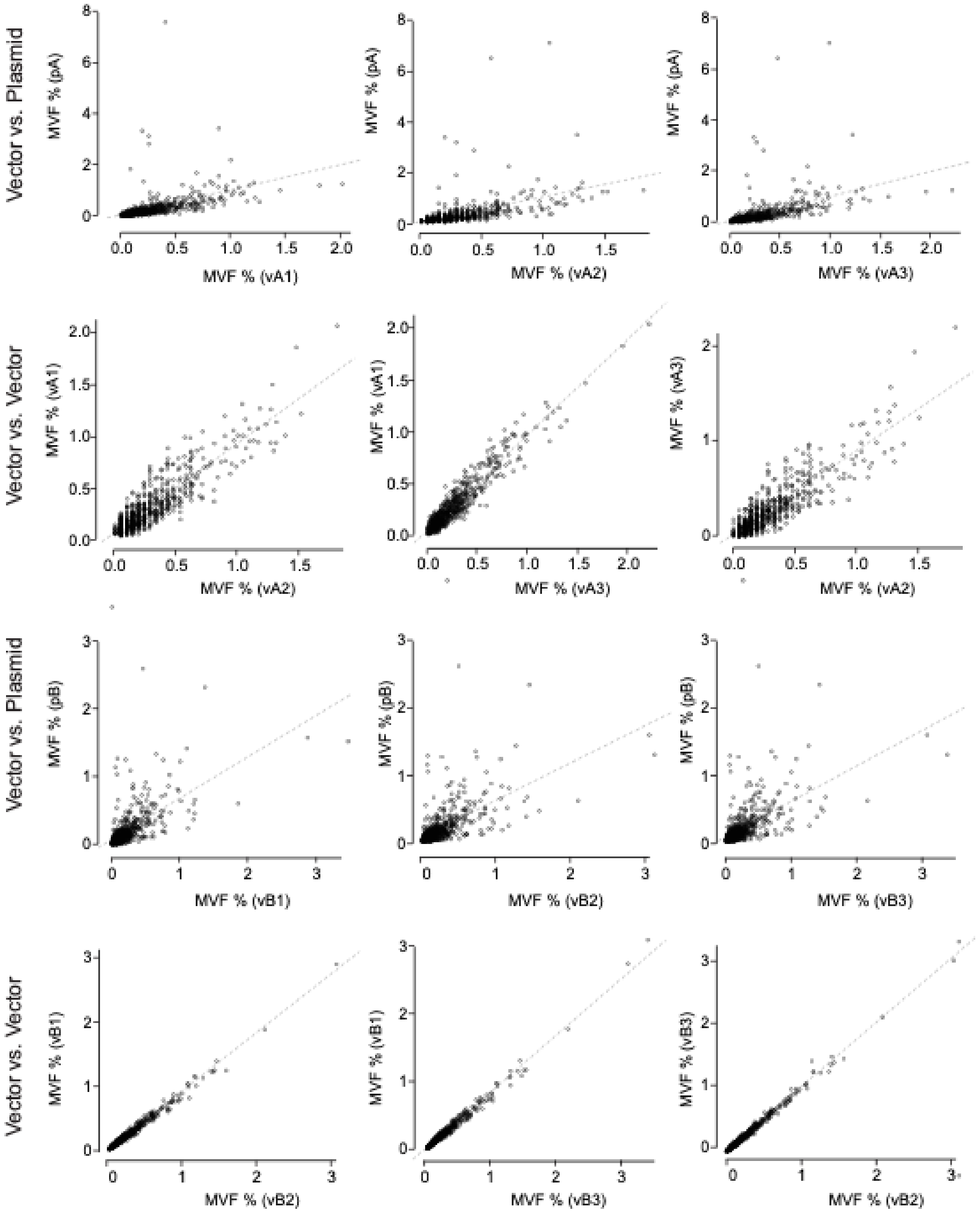

Figure S3. Scatterplot of MVF (%) from ONT between vector and plasmid or between vector lots for Supplier A (top) and Supplier B (bottom). Dots indicate the MVF frequency (%) for each position in the GFP gene. A linear regression is shown as a dotted line. R^2 values are given in the text; all correlations were significant (p<0.0001).

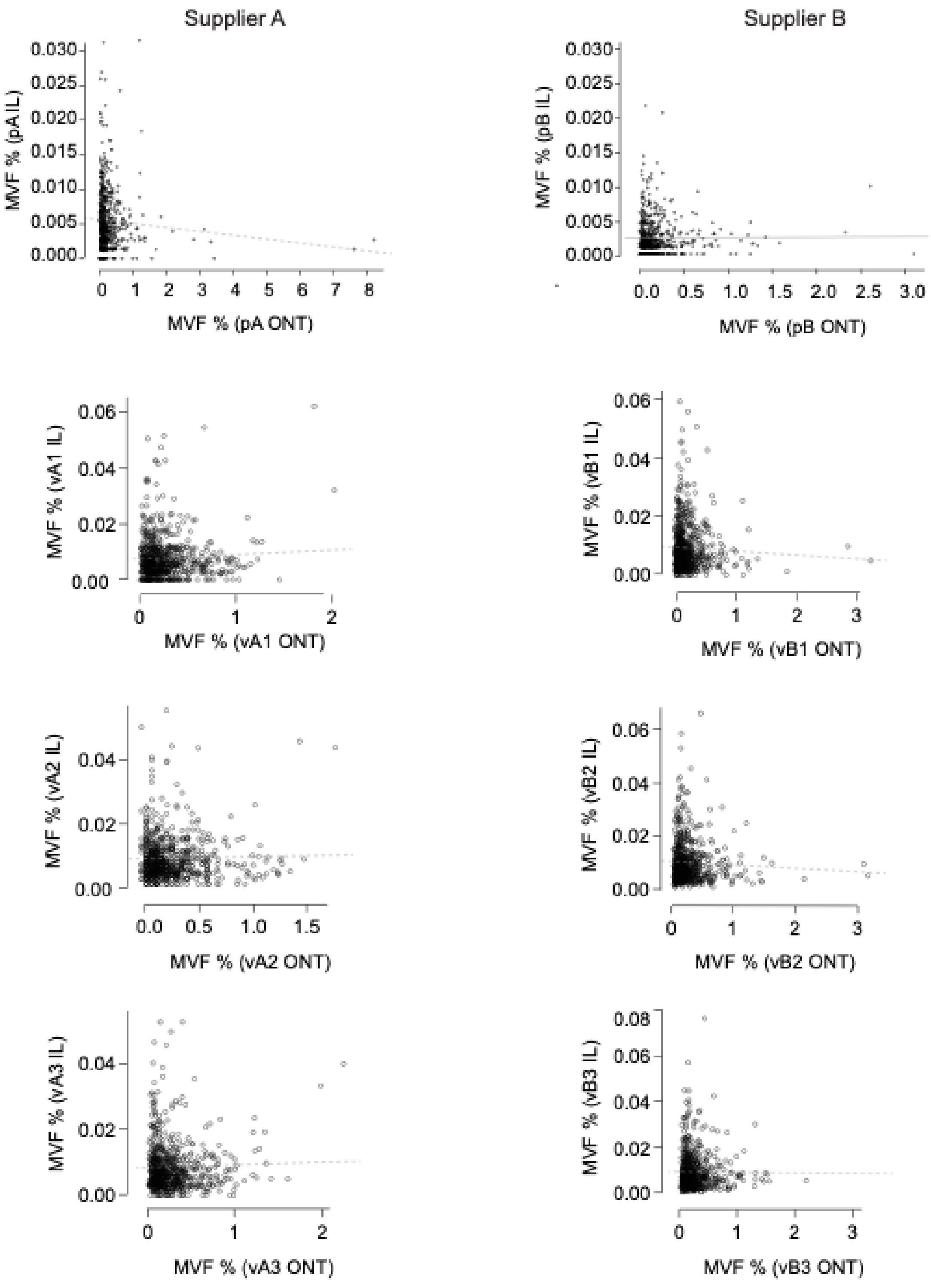

Figure S4. Scatterplot of MVF between Illumina (y-axis) and ONT (x-axis) and for Supplier A (left) and B (right). Dots indicate the MVF frequency (%) for each position in the GFP gene. A linear regression is shown as a dotted line. None of the comparisons were significant (p>0.05).
